# Clinical Features and Severe Outcomes of Depressive Disorders among Healthcare Workers

**DOI:** 10.1192/j.eurpsy.2025.1322

**Published:** 2025-08-26

**Authors:** A. Gaddour, R. Nakhli, A. Brahem, M. Bouhoula, A. Chouchane, R. El Ghzel, H. Kalboussi, M. Moua, O. Maalel, I. Kacem, S. Chatti

**Affiliations:** 1 Department of Occupational Medicine, Faculty of Medicine of Sousse, Ibn Jazzar University Hospital, Tunis; 2 Department of Occupational Medicine, Faculty of Medicine of Sousse, Sousse, Tunisia

## Abstract

**Introduction:**

Depressive disorders among healthcare workers (HCWs) exhibit a range of clinical manifestations, from mild to severe forms, leading to significant consequences such as hospitalization, extended sick leave, and suicide attempts. Understanding these clinical features is essential for the implementation of effective mental health interventions and support services.

**Objectives:**

This study aims to analyze the clinical features of depressive disorders among HCWs, and to assess the relationships between these factors.

**Methods:**

A retrospective descriptive study was conducted over an 11-year period on HCWs in Sousse, Tunisia, who took long-term sick leave for depressive disorders. Data were collected from medical records, and completed with a telephone questionnaire.

**Results:**

Out of 650 cases examined, 48% were classified as having severe depression, while 50% experienced moderate depression, and 2% had mild forms. Anxiety was the predominant clinical feature, occurring in 71.4% of cases and showing a significant correlation with depression severity (p = 0.001). Melancholic features were identified in 33% of the cohort and were also significantly linked to severe depression (p = 0.005). Hospitalization due to depression was necessary for 11.2% of cases, with 4.2% requiring multiple hospitalizations; notably, all hospitalized patients exhibited severe depression (p = 0.001). Additionally, 1.2% of the study population reported a history of suicide attempts, all of whom presented severe depression, although this finding did not attain statistical significance.

**Conclusions:**

This study highlights the importance of understanding the clinical specifications of depressive disorders in HCWs, as severe forms are often associated with hospitalizations and a higher risk of suicide attempts. The results emphasize the need for early intervention and targeted support strategies to address these severe outcomes.

**Disclosure of Interest:**

None Declared

